# Photobiomodulation Therapy in Facial Aesthetic Surgery: A Systematic Review of Efficacy and Safety

**DOI:** 10.1111/jocd.71047

**Published:** 2026-07-10

**Authors:** Leonard Knoedler, Jakob Fenske, Alessia Taggiasco, Romane Gaillard, Guilherme Franceschini Machado, Samuel Knoedler, Theodore Brown, Danni Yang, Kevin Sadati, Thomas Schaschinger, Haizam Oubari, Curtis L. Cetrulo, Alexandre G. Lellouch

**Affiliations:** ^1^ Division of Plastic and Reconstructive Surgery Cedars Sinai Hospital Los Angeles USA; ^2^ Department of Oral and Maxillofacial Surgery Charité—Universitätsmedizin Berlin, Corporate Member of Freie Universität Berlin and Humboldt‐Universität Zu Berlin Berlin Germany; ^3^ Fondazione Policlinico Universitario Campus Bio‐Medico Rome Italy; ^4^ Department of Oral and Maxillofacial Surgery APHP—Assistance Public Des Hôpitaux de Paris Paris France; ^5^ Hospital Santa Marcelina São Paulo Brazil; ^6^ Division of Plastic Surgery, Department of Surgery Yale School of Medicine New Haven Connecticut USA; ^7^ Private Practice Newport Beach California USA; ^8^ Medical Faculty University of Heidelberg Heidelberg Germany; ^9^ Department of Plastic and Reconstructive Surgery Plastic Surgery Research Laboratory, Massachusetts General Hospital, Harvard Medical School Boston Massachusetts USA; ^10^ Department of Plastic Reconstructive and Aesthetic Surgery Hospices Civils de Lyon Lyon France

**Keywords:** clinical global impression of change (CGIC), light‐emitting diode (LED), low‐level laser therapy (LLLT), near‐infrared (NIR), photobiomodulation therapy (PBMT), visual analogue scale (VAS), wound‐healing grade (WH)

## Abstract

**Introduction:**

Advances in facial plastic surgery have increased interest in therapies that expedite recovery and enhance comfort. PBMT, including low‐level laser/light and LED modalities, has been proposed to reduce pain, edema, and ecchymosis and support wound healing after facial procedures. However, clinical adoption is limited by heterogeneous dosing parameters, inconsistent nomenclature, and nonstandardized outcome measures. This study synthesizes clinical evidence on the efficacy, time course, safety, and treatment parameters of PBMT to guide standardization and trial design.

**Methods:**

We conducted a PRISMA 2020‐guided systematic review of PubMed, Embase, Cochrane, and Web of Science for English‐language studies using PBMT after blepharoplasty, rhinoplasty, or facelift with measurable outcomes and parameters. In vitro/cadaver/animal studies, ablative‐laser primary interventions, and non‐original studies were excluded.

**Results:**

The search retrieved 940 articles; after removing 83 duplicates, 857 records were screened and six studies met inclusion criteria. A total of 298 patients were reported, of whom 155 received PBMT; 83 underwent blepharoplasty, 71 nasal surgery, and 1 facelift. PBMT was associated with reduced ecchymosis at POD7, accelerated POR on EBEC composites, POD7 wound‐healing grades, and lower pain/swelling on VAS in periocular and septoplasty cohorts; between‐group rhinoplasty PI was neutral despite within‐group improvement over time. Reporting of parameters and outcome scales was heterogeneous; adverse events were rare.

**Conclusion:**

PBMT appears to reduce pain, edema, ecchymosis, and improve wound healing after facial surgery; however, current evidence is sparse and requires validation in larger cohorts. Standardized protocols and adequately powered blinded trials are needed to confirm efficacy.

**Abbreviations**
EBEC: Erythema Bruising Edema Complete healing blinding assessment trough photos
PBMT: Photobiomodulation Therapy
PI: Primary photogrammetry
POD: Post‐Operative Day
POR: Periocular Recovery

## 1 Introduction

Photobiomodulation (PBM), formerly termed low‐level laser (or light) therapy, is a nonthermal, noninvasive form of phototherapy that utilizes visible and near‐infrared wavelengths to modulate cellular activity and tissue repair. Although PBMT most commonly employs red light (RL, 620–700 nm) and near‐infrared (NIR, 700–1440 nm) wavelengths, owing to their deeper tissue penetration and mitochondrial bioenergetic effects, it can also be delivered using blue [1] (∼400–500 nm) and yellow [2] (∼570–590 nm) light, which are primarily used for more superficial indications such as acne and bacterial colonization (blue light), and erythema modulation or pigmentary/vascular dysregulation (yellow light). Contemporary evidence suggests that PBMT can reduce inflammation, promote angiogenesis, and influence fibroblast behavior and extracellular matrix (ECM) remodeling [3, 4, 5].

Within facial plastic surgery, these photobiological effects are clinically attractive across two broad domains: peri‐operative optimization, including attenuation of pain, edema, ecchymosis, trismus, enhancement of wound healing and scar quality; and dermatologic rejuvenation, including improvements in rhytides, elasticity, and dermal collagen [6, 7]. Early split‐face and controlled trials in aesthetic dermatology reported wrinkle reduction and increased elasticity with red‐light LED therapy [8], findings echoed by more recent randomized studies using red and amber wavelengths and by broader dermatology reviews. In the surgical setting, PBMT has been investigated as an adjunct after procedures relevant to facial plastics. Randomized and controlled studies in oral‐maxillofacial, orthognathic surgery populations and rhinoplasty randomized studies have shown reductions in postoperative pain and edema with PBM, although heterogeneity in dosimetry and protocols tempers certainty [9, 10, 11, 12].

Accordingly, the objective of this systematic review is to synthesize and critically appraise the clinical evidence for PBMT in facial plastic surgery, encompassing perioperative recovery (pain, edema, ecchymosis) and scar outcomes (linear and pathological). By clarifying dose–response relationships and methodological gaps, we aim to inform protocol standardization and define priorities for future randomized trials in this rapidly evolving field.

## 2 Methods

This systematic review adhered to the Preferred Reporting Items for Systematic Reviews and Meta‐Analyses (PRISMA) 2020 guidelines and was registered on PROSPERO 2025 CRD420251156120. A narrative synthesis was chosen due to the heterogeneity in outcome measures, deeming a meta‐analysis unsuitable.

### 2.1 Eligibility Criteria

Eligibility criteria were defined a priori according to the PICOS framework. Eligible studies included English‐language, full‐text original human research. The population consisted of patients undergoing facial plastic/reconstructive/aesthetic procedures (e.g., rhinoplasty, facelift/rhytidectomy, blepharoplasty). The intervention was photobiomodulation (PBM) (low‐level laser/light, red/near‐infrared light, or LED therapy) administered pre‐, intra‐, or postoperatively. Any comparator was accepted, including sham/placebo, standard care, no treatment, or within‐subject controls. In terms of outcomes, studies had to report measurable recovery parameters, such as wound‐healing rate/quality/time, scar quality (preferably via validated scales), patient‐reported outcomes (e.g., pain or satisfaction on VAS), reduction of edema/ecchymosis, and/or adverse events. Eligible study designs included randomized or non‐randomized controlled trials, prospective/retrospective cohorts, case–control studies, and case series/reports; when populations overlapped, the most complete or most recent dataset was selected.

Exclusion criteria were applied to studies that: [1] were in vitro, cadaveric, or animal‐only; [2] used ablative surgical lasers as the primary intervention (e.g., CO_2_ or Er: YAG) rather than PBMT or any non‐red/non–near‐infrared light sources (e.g., blue or yellow light); [3] lacked measurable clinical outcomes; [4] did not report original patient outcome data; or [5] were not available in full text or were not published in English.

### 2.2 Search Strategy and Quality Assessment

We systematically searched PubMed, Embase, Web of Science, and the Cochrane Library from database inception to *September 5, 2025*, using the following search string using free key words:

((*photobiomodulation* OR “low‐level laser therapy” OR “low‐level light therapy” OR *“LLLT”* OR “red light therapy” OR “light‐emitting diode therapy” OR *“near‐infrared therapy”* OR *“LED therapy”* OR *“laser therapy”*) AND (*blepharoplasty* OR *rhinoplasty* OR *facelift*)).

To assess the quality of the included studies, the Newcastle‐Ottawa Scale (NOS) and the Level of Evidence (LOE) were applied, outlined in Tables S1 and S2. The NOS evaluated three key domains, assigning up to nine stars to each study: (1) selection of study cohorts (maximum of four stars), (2) comparability of groups (maximum of two stars), and (3) assessment of outcomes/exposures (maximum of three stars).

Two authors (A.T. and G.F.M.) independently screened all titles and abstracts using Rayyan v1.6 (Cambridge, Massachusetts). Screening, baseline study characteristics and outcome data were extracted independently, with discrepancies resolved through consultation with the first (L.K.) and senior author (A.G.L.). Full search terms are provided in Table S3.

### 2.3 Data Extraction

During the blinded, dual‐review process the following variables were extracted: first author, population size (PBMT‐treated vs. control), age range, surgical procedure or reason for treatment (e.g., blepharoplasty, rhinoplasty, facelift), associated procedures, timing of PBMT intervention (pre−/intra−/post‐operative and postoperative day), device parameters (power density in mW/cm^2^, fluence in J/cm^2^, wavelength in nm, exposure duration), treatment schedule (session frequency and total sessions), timing of follow‐up assessments, outcome instruments and their primary readouts, adverse effects, and overall results as seen in Table 1.

**TABLE 1:** General study characteristics.

First author et al.	Study design	Country	Population size (HF or R)	Age range	Surgical Procedure/Reason for treatment	Associated procedure	Timing of intervention	Power Density (mW/cm^2^)	Fluence (J/cm^2^)	Wavelength (nm)	Duration	Frequency of Sessions (Follow up)	Total Sessions
Jamalpour M. et al. 2025	RCT, DB	Iran	20 (R)	N/A	Post‐rhinoplasty swellingness	N/A	POD 0‐7‐9‐11‐17‐20‐24‐27‐31‐34‐38‐41‐45‐48‐52‐55 day	44 mW/cm^2^	4 J/cm^2^ per area	940 nm	90 s per area, ~20 min/session	8 weeks (8 POW)	16
Ye H et al. 2025	Prospective, single center, SB‐RCT	China	145 (R)	N/A	Incisional blepharoplasty	N/A	POD 1, 2, 3, and 7 days	65 mW/cm^2^	60 J/cm^2^ per session	830 nm	16 min 23 s per session	Daily on POD1–3, then once on POD7 (1–4 POW)	4
Kastyro et al. 2021	Non‐Randomized comparative study	Russia	62 (HF)	18–44	Septoplasty	N/A	POD 3‐6‐24‐48 h	N/A	N/A	890 nm (pulsed IR) for early sessions; 630 nm (red, continuous modulated) intranasally at 48 h	2 min per session	Three sessions in first 24 h 3 h, 6 h, 24 h, then one session at 48 h (2‐POD)	4
Karimi et al. 2020	RCT, SB	Iran	60 (R)	20–43	Post‐Rhinoplasty, ecchymosis.	N/A	POD 1–30 days	N/A	N/A	660 nm (red SLD array), 840 nm (IR SLD array), 830 nm (IR laser probe)	5 s pulse duration	One session comprising a three‐step protocol 660, 840, 830 nm, (30‐POD)	1
Barolet el al 2010	Case series	Canada	3 (HF)	27–57	Facelift, Abnormal scars (hypertrophic/keloid)	Surgical excision or CO_2_ (laser resurfacing), PBMT prophylaxis on one side.	POD 1–30 days	30 mW/cm^2^	27 J/cm^2^ per session	805 nm	15 min per session.	Once daily for 30 days (77‐POW)	30
Trelles et al. 2006	Case series	Spain	10 (HF)	44–59	Upper/lower blepharoplasty for wound healing, erythema, edema	Combined Er:YAG/continuous‐wave CO_2_ laser resurfacing	POD 1‐3‐7‐10 days	80 mW/cm^2^	96 J/cm^2^ per session	633 ± 3 nm	20 min per session	4 sessions total over the first postoperative week (6‐POW)	4

Abbreviations: DB, double‐blind; HF, hemi‐face; LED, light‐emitting diode; LLLT, low‐level laser therapy; NIR, near‐infrared; PBMT, photobiomodulation therapy; POD, post‐operative day; POW, post‐operative week; RCT, randomized controlled trial; SB, single‐blind.

## 3 Results

### 3.1 Study Selection and Characteristics

**FIGURE 1:**
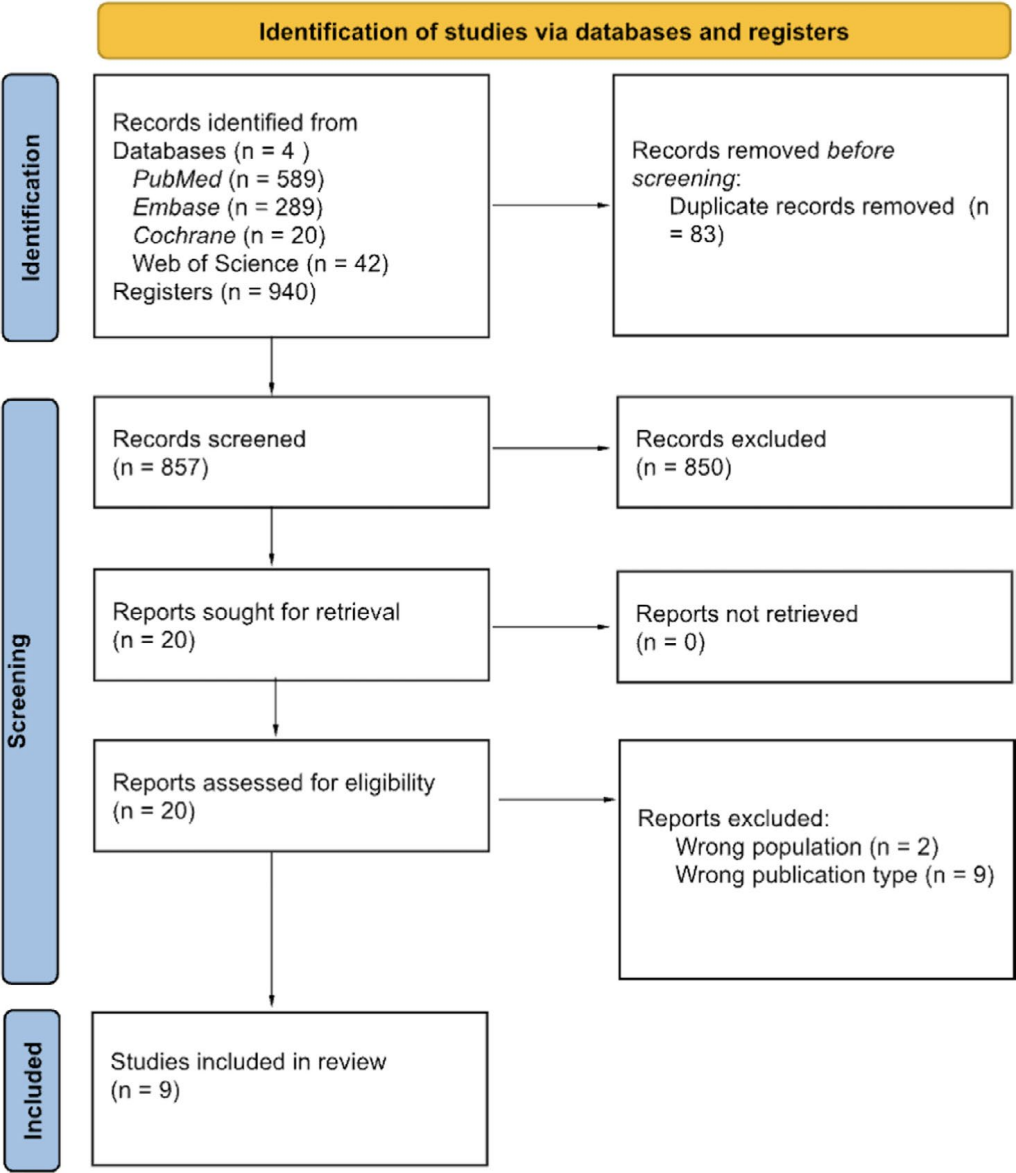
PRISMA 2020 flowchart.

**FIGURE 2:**
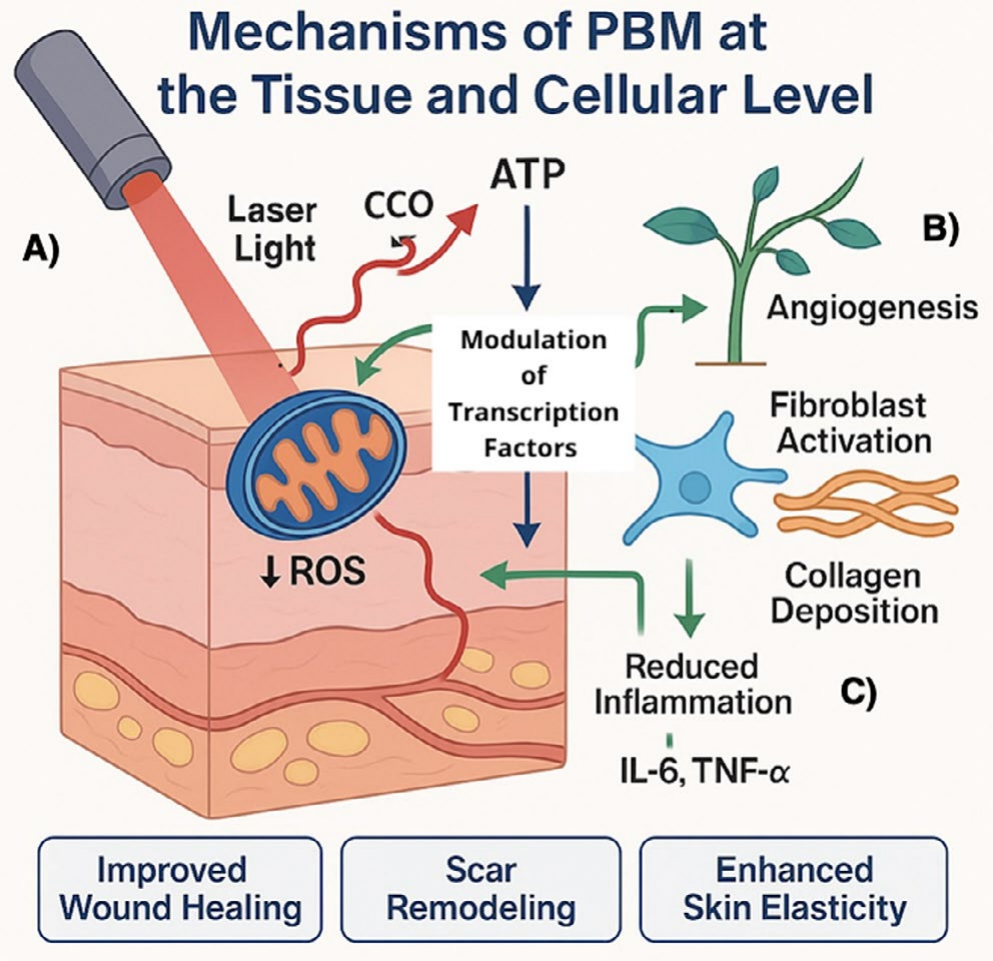
Mechanisms of photobiomodulation (PBM) at the tissue and cellular level. (A) Laser/light irradiation is absorbed by mitochondrial cytochrome c oxidase (CCO), leading to increased ATP production and reduced reactive oxygen species (ROS), with downstream modulation of transcription factors. (B) These signaling changes promote angiogenesis and fibroblast activation, supporting collagen deposition and extracellular matrix remodeling. (C) PBMT also reduces inflammation, including decreased pro‐inflammatory cytokines (IL‐6, TNF‐α), resulting in improved wound healing, scar remodeling, and enhanced skin elasticity.

As outlined in Figure 1, the initial search identified 940 records across databases: PubMed (589), Embase (289), Cochrane [5], and Web of Science (42). Included studies evaluated PBMT, the proposed cellular and tissue‐level mechanisms of which are summarized in Figure 2. After removal of 83 duplicates, 857 unique records remained for title and abstract screening. Most were excluded as irrelevant to perioperative PBMT in facial plastic surgery.

### 3.2 Distribution of PBMT Use by Surgery Type and Complication

Among the 155 [12, 13, 14, 15, 16, 17] patients who underwent photobiomodulation therapy (PBMT), the most frequently reported indication, as outlined in Table 2, was postoperative swelling or edema, observed in 93 patients (60.0%) [18]. Wound healing was the second most common reason, reported in 83 patients (53.55%) [15, 18], followed by pain management in 41 patients (26.45%) [16, 18] and treatment for postoperative ecchymosis in 40 patients (25.81%) [13, 18]. PBMT was least frequently applied for abnormal scar management, involving 1 patient (0.65%) [14]. In one study [18], PBMT was administered to 10 patients to enhance postoperative wound healing and to reduce edema, erythema, bruising, and pain. Because some studies, such as this one, reported overlapping therapeutic objectives, the totals per category are not mutually exclusive and represent the frequency of each indication across all patients rather than distinct individuals. In Barolet 2020, two additional patients were excluded from the analysis as PBMT was used for indications outside the scope of this review, specifically, for acne scars in one case and for a hypertrophic scar secondary to surgical removal of nevi in the areolar region in another.

**TABLE 2:** Common postoperative indications for photobiomodulation therapy (PBMT) across included studies.

	EC	AS	W	SW	P
Population	40	1	83	93	41
Mean (%)	40/155 (26)	1/155 (0.6)	83/155 (53)	93/155 (60)	41/155 (26)

Abbreviations: AS, abnormal scar; EC, ecchymosis; P, pain; SW, swelling; W, wound healing.

By procedure category, as outlined in Table 3, PBMT was most commonly applied after blepharoplasty, accounting for 83 of 155 patients (53.55%) [15, 18]. The remaining cases comprised 71 rhinoseptoplasty procedures (45.80%) [12, 13, 16] and 1 facelift case (0.65%) [14].

**TABLE 3:** Most common procedures utilizing post‐operative photobiomodulation therapy (PBMT).

	RH/SP	BP	Facelift
Population	71	83	1
Mean (%)	71/155 (46)	83/155 (53)	1/155 (0.6)

Abbreviations: BP, blepharoplasty; RH/SP, rhinoplasty/septoplasty.

### 3.3 Objective Post‐Operative Outcomes

Depending on the outcome scale applied, as outlined in Table 4, four major domains were assessed to determine the effects of PBMT: post‐operative ecchymosis, scar quality (including reductions in height and surface area), edema, and wound‐healing progression.

**TABLE 4:** Scales of outcome measures across included studies on photobiomodulation therapy (PBMT) patients.

	Ecchymosis	Scar assessment		Edema	Wound healing	
	EG	VSS	CGIC	PI	EBEC	WH
PMBT‐P (*n* control)	30 (30)	3 (3 HF)	3 (3HF)	10 (10)	10 (10 HF)	73 (72)
*p‐*value (POD/POW)	0.005 (7d)	N/A	N/A	Between groups: *p* > 0.05 (1, 4, or 8 weeks) Within‐group, tip rotation: *p* = 0.008, 0.003, 0.005 (1, 4, 8 weeks) (*p* = 0.008, 0.003, 0.005); Within group nasal width: *p* = 0.048, 0.016 (1 & 4 weeks)	< 0.0001	0.003 (7d)
Session	1	30	30	16	4	4
Result	A single PBMT session on POD1 (red 660 nm + IR 840/830 nm) significantly reduced periorbital ecchymosis at 7 days versus control, with no complications	VSS decreased more on PBMT sides (−77.8%, −36.4%, −12.5%) than controls (−11.1%, −28.6%, 0%), favoring PBMT	CGIC favored in all cases (PBMT scores 4/3/2 vs. control 1/1/1)	No between‐group superiority. Meaningful within‐group improvements in tip rotation and transient narrowing—suggesting edema reduction.	PMBT resolved significantly faster: erythema 3.7 vs. 7.6 days (*p* < 0.0001); bruising 4.7 vs. 8.5 (*p* < 0.0001); edema 3.7 vs. 7.6 (*p* < 0.0001); complete re‐epithelialization 13.5 vs. 26.8 days (*p* < 0.0001)	PBMT improved Day‐7 wound healing effectiveness (higher Grade‐A rate)

Abbreviations: CGIC, global impression of change (0–4); d, day; EBEC, erythema bruising edema complete healing (blinded assessment through photos); EG, ecchymosis grading (0–4); HF, hemi‐face; PBMT‐P, photobiomodulation therapy patients; PI, primary photogrammetry; POD, postoperative day; PP, PRIMOS 3D profilometry; VSS, Vancouver scar scale; WH, wound healing Grade (0–4).

For postoperative ecchymosis, one study [13] evaluated bruising using the Ecchymosis Grading Scale (EG, 0–4) following a single PBMT session administered on postoperative day 1 with combined red and infrared wavelengths at 660 nm plus infrared 840/830 nm. By postoperative day 7, EG scores were significantly lower in the PBMT group, indicating faster clearance of periorbital bruising without reported complications.

For scar assessment, another study used the validated Vancouver Scar Scale (VSS) and the blinded Clinical Global Impression of Change (CGIC, 0–4) to evaluate treatment response [14]. PBMT‐treated areas demonstrated greater improvement than paired controls, with marked reductions in overall VSS scores. Objective surface profiling revealed a significant decrease in scar height during the early postoperative period, whereas control areas showed no significant change. The blinded CGIC scores likewise favored PBMT.

Edema reduction was analyzed using two distinct approaches capturing swelling dynamics over time: photogrammetry [12] and blinded clinical photography [18] assessment. In the first study, the PBMT arm showed significant within‐group reductions in swelling from postoperative Week 1 through Week 8, as reflected by decreased nasal width and increased tip rotation. No significant between‐group differences were detected at early or late follow‐up points. In the second study, the PBMT‐treated sides showed significantly faster resolution of edema. Erythema, bruising, and re‐epithelialization were also assessed through blinded clinical photography and showed significant differences. The PBMT‐treated sides showed a faster resolution of all parameters, resolving within approximately 3–5 days, and full re‐epithelialization achieved by around two weeks postoperatively, nearly halving the recovery time compared with controls.

**FIGURE 3:**
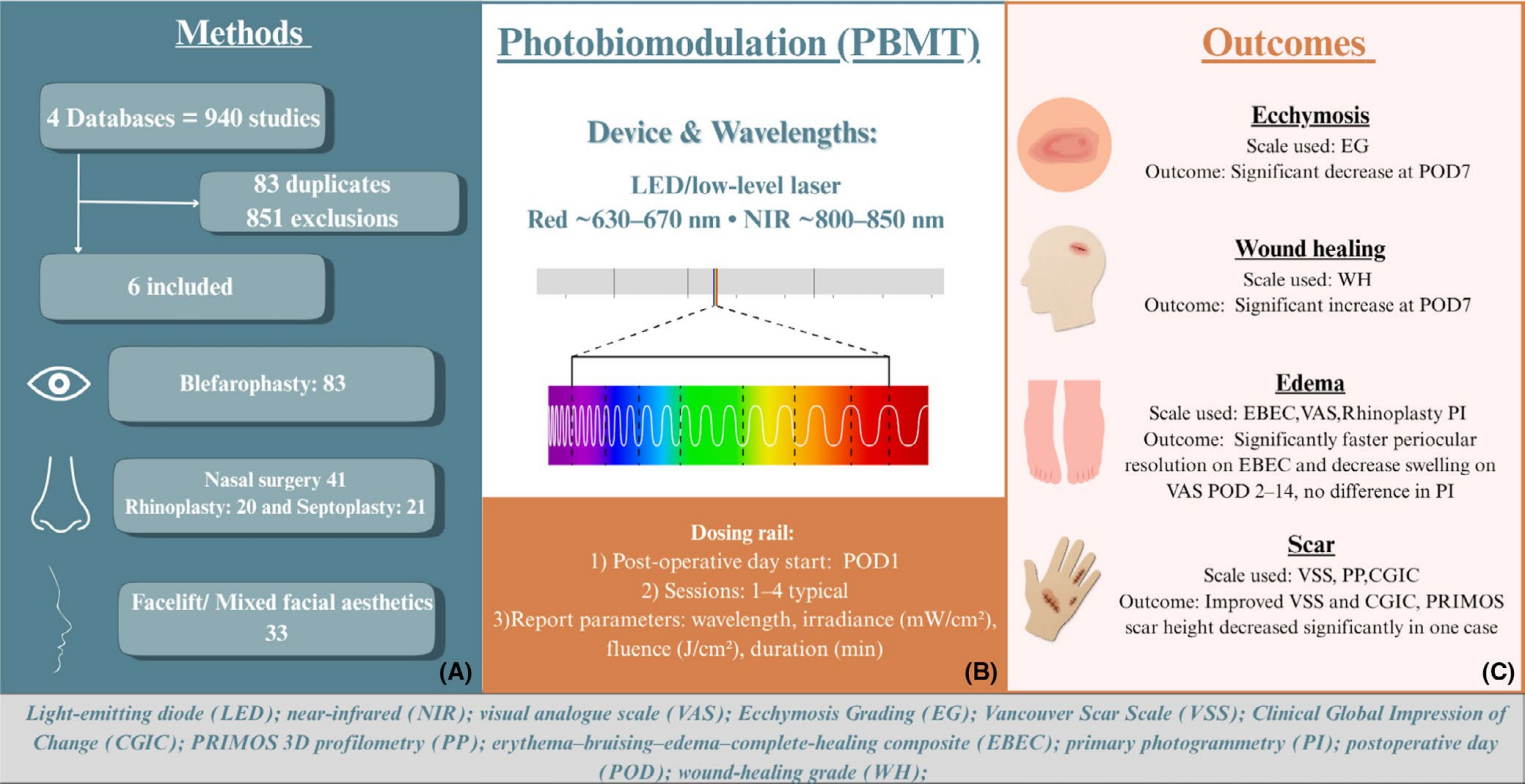
Overview of included studies, PBMT parameters, and postoperative outcomes. (A) Study selection process and distribution of included procedures across the 4 databases screened, summarizing duplicates/exclusions and the final included studies, with procedure categories (blepharoplasty, nasal surgery—rhinoplasty/septoplasty, and facelift/mixed facial aesthetics). (B) Photobiomodulation therapy (PBMT) characteristics, including device type (LED/low‐level laser) and wavelength ranges (red ~630–670 nm; near‐infrared [NIR] ~800–850 nm), and commonly reported dosing features (postoperative day start, number of sessions, and key parameters: Wavelength, irradiance, fluence, and duration). (C) Clinical outcomes assessed after facial aesthetic surgery—ecchymosis, wound healing, edema, and scar—along with the scales used (EG, WH, EBEC, VAS, PI, VSS, PP, CGIC) and a summary of the main findings reported across studies.

Regarding specifically wound healing, besides blinded clinical photography assessment, another study [15] conducted an investigation assessing early repair using a wound‐healing scale (WH, 0–4) after blepharoplasty. The study reported significantly higher healing scores by postoperative Day 7 in the PBMT group, consistent with accelerated early tissue recovery following a short postoperative treatment course. A summary of the scales used and key findings for each domain is presented in Figure 3.

### 3.4 Patient‐Reported Outcomes

Pain assessment was primarily conducted using the validated 0–10 visual analogue scale (VAS), as outlined in Table 5. Significant reductions in pain were observed as early as postoperative Day 1, with continued improvement during the early postoperative period. In some reports, these differences persisted from postoperative Day 2 through Day 14, indicating a sustained analgesic effect. Overall, PBMT‐treated areas demonstrated both lower pain intensity in the first 24 h and faster resolution of discomfort over time.

**TABLE 5:** Visual analogue scale throughout included studies.

First author et al.	Population treated with PBMT (*n* control)	Reason for VAS	*p* (POD)	Session of PBMT	Result	Population treated with PBMT (*n* control)
Ye H et al. 2025	73 (72)	Periocular swelling	*p* < 0.001 (1–14)	4	PBMT significantly reduced eyelid swelling from POD2 onward and at all weekly follow‐ups (1–4 weeks) vs. control.	73 (72)
	73 (72)	Pain	*p* < 0.001 (2–14)	4	PBMT produced significantly lower pain from POD2 through Week 2 vs. control.	73 (72)
Kastyro et al. 2021	31 (30)	Pain	*p* < 0.001 (1)	3	PBMT significantly reduced early post‐op pain (notably 6–24 h)	31 (30)
Karimi et al. 2020	30 (30)	Ecchimosis severity	0.64 (7)	1	PBMT did not significantly change patient‐reported VAS (0–10) for ecchymosis at Day 7 vs. control, although both groups improved from Day 1 to Day 7	30 (30)
Trelles et al. 2006	10 (10 hemi‐face)	Pain	*p* < 0.001 (1)	4	PBMT side had much lower pain (mean VAS 2.9 vs. 6.9) and faster pain resolution (1.1 h vs. 2.9 h)	10 (10 hemi‐face)

Abbreviations: PBMT, photobiomodulation therapy; POD, post‐operative day; VAS, visual analogue scale (0–10).

Although the VAS is standardized for pain evaluation, several studies also used it to assess ecchymosis and swelling severity. Bruise severity was assessed at postoperative Day 7 and found no significant difference between PBMT and control, although both groups showed gradual improvement from postoperative Day 1 to Day 7 [13]. Regarding swelling, significant reduction of periocular edema was observed beginning on postoperative Day 2, with improvements remaining evident at all follow‐ups through the second postoperative week [15].

Taken together, while patient‐perceived bruising intensity may not differ significantly at a single later time point, PBMT is associated with earlier and more pronounced reductions in pain and swelling, with significant effects emerging within the first one to two postoperative days and persisting throughout the initial 2 weeks of recovery.

## 4 Discussion

Photobiomodulation therapy (PBMT), which includes low‐level laser and LED‐based modalities, has emerged as a potential perioperative adjunct in facial plastic surgery, aiming to accelerate recovery and improve patient comfort. In the context of rhinoplasty, blepharoplasty, and facial surgical procedures, PBMT is applied to mitigate early morbidity (edema, ecchymosis, pain) and to support tissue repair and scar quality, with outcomes captured on standardized scales (e.g., VSS, VAS). Our objective is to contextualize the magnitude and consistency of effect, identify methodological gaps that limit comparability, and outline priorities for protocol standardization and adequately powered trials to guide evidence‐based use of PBMT in facial plastic surgery.

### 4.1 Clinical Applications and Limitations of PBMT in Facial Plastic Surgery

PBMT was most frequently applied following blepharoplasty, which accounted for 83 of 155 patients (53.55%) [15, 18]. This predominance suggests a particular research focus on periorbital applications of PBMT. Rhinoseptoplasty were the next most common procedures, involving 71 patients (45.80%) [12, 13, 16], while facelift cases were notably underrepresented, with only one reported instance (0.65%) [14]. Overall, the sample sizes for blepharoplasty and rhinoplasty were relatively comparable.

Only a small fraction of the screened studies (6 out of 940) met our inclusion criteria. This limited yield is likely attributable to a combination of study‐level and field‐level factors. Previous research has highlighted a persistent lack of consensus on key PBMT parameters, such as wavelength, power density, fluence, and pulsing, which contributes to substantial heterogeneity and limits cross‐study comparability [19]. This variability was also evident among the included studies, where power densities ranged from 30 to 80 mW/cm^2^, fluences from 4 to 96 J/cm^2^, and treatment durations from 5 s to 20 min, typically delivered over multiple postoperative sessions.

To reduce heterogeneity, we included only studies using red or near‐infrared PBMT wavelengths as it aligns with prior reviews identifying red (~630–700 nm) and near‐infrared (~780–950 nm) as the main therapeutic range in cutaneous and perioperative protocols. Restricting to this range helped limit parameter‐related confounding and avoided bias from comparing different photobiological mechanisms (e.g., blue or yellow light) settings [5, 20].

Moreover, the field's terminology and conceptual framework have evolved over time, from “low‐level laser therapy (LLLT)” to “photobiomodulation” and earlier skepticism, coupled with inconsistent nomenclature, likely hindered the indexing and retrieval of relevant studies [21]. These methodological, systemic, and historical factors together contribute to the limited number of studies meeting the strict inclusion criteria of this review.

### 4.2 Time Related Outcome

Across included studies, PBMT was consistently initiated early in the postoperative period, with clinical effects emerging over the first postoperative days, suggesting a time‐dependent response. When initiated within the first postoperative week, it was shown in a randomized study of 10 patients, how PBMT may accelerate the trajectory of healing across sequential milestones: although no difference was evident on POD1 (e.g., edema comparable between sides), clinically meaningful changes were noticeable by POD3 and 4, with edema and erythema resolving on the treated side roughly two times faster than the control. In the same study, trough a blinded photographic comparison, also bruising likewise cleared sooner, at POD5, 3 days faster than the control, suggesting an early anti‐edematous and microvascular stabilization effect. This temporal advantage culminated in substantially earlier closure marked by complete re‐epithelization by POD 13 effectively shortening convalescence by about 2 weeks [12]. In another cohort study of 73 patients, at POD7, incision appearance was reduced on standardized wound‐healing grades, consistent with more orderly tissue remodeling. Taken together, these time‐anchored outcomes indicate that PBMT's benefits are timing‐dependent: early postoperative initiation appears motivating the conversion from a transient inflammatory stage into faster structural repair, with clinically visible gains emerging within days and consolidating over the first two to three postoperative weeks [15, 18]. Concordant findings in adjacent fields reinforce its utility in the early time course: oral‐maxillofacial trials and reviews report pain and edema reductions associated with PBMT within 24–72 h after third‐molar surgery [10, 21, 22] (although effect sizes vary with parameters and study quality). However, no studies evaluated delayed initiation of PBMT, precluding direct comparison of early versus late treatment timing.

Mechanistically, experimental data suggest that PBMT attenuates this early inflammatory surge by reducing NF‐κB–mediated cytokine output and shifting macrophages toward an M2, pro‐repair profile, a regulatory shift that may help explain the earlier resolution of edema and ecchymosis observed within the first 3–5 postoperative days [23]. Early red‐light PBMT may exert its most meaningful effects during the immediate postoperative period, when inflammatory activity is at its peak and strongly influences the tempo of recovery. Concurrent mitochondrial photostimulation and photorelease of nitric oxide improve microvascular perfusion, supporting keratinocyte and fibroblast activity at a stage when cellular energy demand is highest [5, 23, 24]. In parallel, PBMT has been shown to upregulate pro‐healing pathways, including VEGF/HIF‐1α that would provide a biologic rationale for the accelerated re‐epithelialization noted by approximately postoperative Day 7 [25]. Taken together, these temporally aligned effects offer a coherent mechanistic framework for the earlier transition from inflammation to proliferation.

### 4.3 Patient Related Outcome

Across trials, PBMT yielded earlier and larger patient‐perceived benefits, with clear effect sizes where data permit. In a split‐face study, time to pain relief was twice as fast with PBMT (1.1 h vs. 2.9 h), with a ~62% shorter time to resolution, and with a peak in pain intensity of ~58% lower (mean VAS 2.9 vs. 6.9) [18]. These gains emerged within the first 24 h, aligning with another study who also observed clinically meaningful pain reductions over 6–24 h [16]. In a separate trial, of 73 patients receiving PBMT, it was also reported lower postoperative anxiety by the end of the first week, suggesting that its benefits extend beyond analgesia to broader aspects of subjective recovery and well‐being.

These VAS findings align with contemporary literature where a 2024 systematic review found that PBMT significantly improved both healing indices and VAS pain scores compared to controls in the early postoperative period, reinforcing a rapid‐onset effect profile [21]. Earlier work similarly concluded that PBMT is an appropriate modality for reducing postoperative pain without safety concerns, consistent with patient‐reported benefit on VAS [26]. Photobiomodulation therapy appears to provide rapid pain relief, with additional improvements in perceived recovery and patient satisfaction. Future studies should standardize VAS timing and control concurrent analgesic use to better quantify effects.

### 4.4 Limitations

This review is limited by the small number of post‐surgical PBMT studies in facial plastic surgery, typically small samples, and generally lower‐level designs. The restriction to English‐language publications may have excluded relevant studies published in other languages, representing a potential source of selection bias. Terminology (“LLLT” vs. “PBMT”), device parameters (wavelength, irradiance, fluence, timing, dose), and outcome measures/timepoints are highly variable (EG, VSS, PRIMOS, EBEC, VAS, and WH), reducing comparability and limiting dose–response conclusions. Included wavelengths (630–940 nm) fall within the accepted therapeutic window and were well tolerated without reported adverse effects, but co‐interventions and incomplete blinding increase bias risk, particularly for VAS pain scores. Follow‐up is usually short, safety reporting inconsistent and generalizability limited by heterogeneous devices and settings. Future work should use standardized protocols, fixed assessment timepoints (e.g., POD2, POD7, Week 2), adequately powered blinded designs, and systematic long‐term safety/outcome reporting.

## 5 Conclusion

Photobiomodulation therapy (PBMT) shows meaningful potential as an adjunct to improve postoperative recovery in facial aesthetic surgery, with early reductions in pain, swelling, ecchymosis, and accelerated wound healing reported across available studies. Yet, variability in treatment parameters (wavelength, fluence, irradiance, and session frequency), inconsistent timing of initiation, heterogeneous outcome measures, and mixed terminology (LLLT vs. PBM) limit cross‐study comparability and reduce confidence in dose–response interpretation. The field would benefit from standardized, procedure‐specific PBMT protocols defining optimal parameter ranges, uniform postoperative assessment timepoints, and core outcome sets. The limited number of included studies and their small cumulative sample size underscore the need for larger, adequately powered trials to substantiate these preliminary findings and support broader clinical adoption in aesthetic practice.

## Author Contributions

L.K. and A.G.L. conceived the study. L.K., J.F., and A.G.L. designed the review methodology. L.K., A.T., G.F.M., conducted the literature search, study screening, and data extraction. R.G., S.K., and A.T., T.B., D.Y., analyzed and synthesized the data and drafted the manuscript., K.S., T.S., and H.O. critically reviewed the manuscript and contributed to interpretation and presentation of findings. C.L.C. and A.G.L. provided senior oversight, feedback and ensured methodological rigor. All authors read and approved the final manuscript.

## Funding

The authors have nothing to report.

## Conflicts of Interest

The authors declare no conflicts of interest.

## Supporting information


**Table S1:** Study rating based on level of evidence.
**Table S2:** Study rating based on the Newcastle‐Ottawa‐scale.
**Table S3:** Full search string for each database included.

## Data Availability

The data that support the findings of this study are available in Public domain literature databases at https://pubmed.ncbi.nlm.nih.gov/. These data were derived from the following resources available in the public domain: PubMed, Embase and Cochrane Library.
