# Supplementary material for: Photobiomodulation Therapy in Facial Aesthetic Surgery: A Systematic Review of Efficacy and Safety
Source: J Cosmet Dermatol. 2026 Jul 10;25(7):e71047. doi: 10.1111/jocd.71047 (PMC13354859; doi:10.1111/jocd.71047)
Supplement: Supplementary file 1 — Table S1: Study rating based on level of evidence. Table S2: Study rating based on the Newcastle‐Ottawa‐scale. Table S3: Full search string for each database included. [file JOCD-25-e71047-s001.docx]

**Supplementary Digital Content**

**Supplementary table 1.** Study rating based on level of evidence.

| **Author** | **DOI** | **Study design** | **LE** |
| --- | --- | --- | --- |
| Ye H et al 2025 | 10.1007/s00266-024-04374-7 | Single-centre, prospective, single-blind randomized controlled trial | I |
| Jamalpour M. et al 2025 | 10.1016/j.pdpdt.2025.104723 | Randomized, double-blind, controlled trial | II |
| Karimi et al 2020 | 10.1007/s00266-020-01760-9 | Randomized, single-blind, controlled trial | II |
| Kastyro et al 2021 | 10.1134/S1607672921050112 | Non randomized comparative study | III |
| Barolet el al 2010 | 10.1002/lsm.20952 | Case series | IV |
| Trelles 2006 | 10.1080/14764170600607731 | Case series | IV |

DOI, Digital Object Identifier; LE, Level of evidence

**Supplementary table 2.** Study rating based on the Newcastle-Ottawa-scale

| **Author** | **Selection**  **(Max 4)** | **Comparability**  **(Max 2)** | **Outcome**  **(Max 3)** | **Total**  **(Max 9)** |
| --- | --- | --- | --- | --- |
| Karimi et al 2020 | 2 | 2 | 2 | 6 |
| Jamalpour M. et al 2025 | 2 | 2 | 2 | 6 |
| Ye H et al 2025 | 2 | 2 | 2 | 6 |
| Kastyro et al 2021 | 2 | 2 | 2 | 6 |

**Supplementary table 3.** Full search string for each database included

| **Database** | **Search String** | **Results** |
| --- | --- | --- |
| PubMed | ((photobiomodulation OR "low-level laser therapy" OR "low-level light therapy" OR "LLLT" OR "red light therapy" OR "light-emitting diode therapy" OR "near-infrared therapy" OR "LED therapy" OR "laser therapy") AND (blepharoplasty OR rhinoplasty OR facelift)) | 589 |
|  |  |  |
| Embase | ((photobiomodulation OR "low-level laser therapy" OR "low-level light therapy" OR "LLLT" OR "red light therapy" OR "light-emitting diode therapy" OR "near-infrared therapy" OR "LED therapy" OR "laser therapy") AND (blepharoplasty OR rhinoplasty OR facelift)) | 289 |
|  |  |  |
| Web of Science | ((photobiomodulation OR "low-level laser therapy" OR "low-level light therapy" OR "LLLT" OR "red light therapy" OR "light-emitting diode therapy" OR "near-infrared therapy" OR "LED therapy" OR "laser therapy") AND (blepharoplasty OR rhinoplasty OR facelift)) | 42 |
|  |  |  |
| Cochrane Library | ((photobiomodulation OR "low-level laser therapy" OR "low-level light therapy" OR "LLLT" OR "red light therapy" OR "light-emitting diode therapy" OR "near-infrared therapy" OR "LED therapy" OR "laser therapy") AND (blepharoplasty OR rhinoplasty OR facelift)) | 20 |
